# Iron Supply of Multivitamins–Multiminerals Commercialized Online by Amazon in Western and Southern Europe: A Labeling Analysis

**DOI:** 10.3390/nu16183140

**Published:** 2024-09-17

**Authors:** Margherita G. M. Mattavelli, Giacomo Piccininni, Gabriel F. Toti, Mario G. Bianchetti, Luca Gabutti, Sebastiano A. G. Lava, Carlo Agostoni, Pietro B. Faré, Gregorio P. Milani

**Affiliations:** 1 Pediatric Institute of Southern Switzerland, Ente Ospedaliero Cantonale, 6500 Bellinzona, Switzerland; margherita.mattavelli@gmail.com (M.G.M.M.); giacomopiccininni@gmail.com (G.P.); 2 Family Medicine Institute, Faculty of Biomedical Sciences, Università della Svizzera Italiana, 6900 Lugano, Switzerland; gatoti90@gmail.com (G.F.T.); mario.bianchetti@usi.ch (M.G.B.); luca.gabutti@eoc.ch (L.G.); 3 Pediatric Cardiology Unit, Department of Pediatrics, Centre Hospitalier Universitaire Vaudois and University of Lausanne, 1011 Lausanne, Switzerland; webmaster@sebastianolava.ch; 4 Pediatric Unit, Fondazione IRCCS Ca’ Granda Ospedale Maggiore Policlinico, 20122 Milan, Italy; carlo.agostoni@unimi.it; 5 Department of Clinical Sciences and Community Health, Università degli Studi di Milano, 20122 Milan, Italy; 6 Department of Infectious Diseases, University Hospital Zurich, 8091 Zurich, Switzerland; pietro.fare@usz.ch

**Keywords:** Amazon, multiminerals, multivitamins, iron

## Abstract

**Background.** In high-income countries, shopping for non-prescription multivitamin–multimineral supplements has tremendously increased. **Objective and Methods.** The purpose of this labeling analysis is to inform on the daily elemental iron (with or without vitamin C) supply provided by multivitamin–multimineral supplements sold online by Amazon in Western and Southern Europe (amazon.es^®^, amazon.de^®^, amazon.it^®^, and amazon.fr^®^). **Results.** We identified 298 iron-containing multivitamin–multimineral preparations sold by Amazon marketplaces: 153 preparations sourced from amazon.de^®^, 68 from amazon.fr^®^, 54 from amazon.it^®^, and 23 from amazon.es^®^. The daily iron dose provided by these preparations was 14 [5–14] mg (median and interquartile range), with no differences among the marketplaces. Approximately 90% (n = 265) of the preparations contained ferrous iron. Moreover, 85% (n = 253) of the preparations were fortified with vitamin C in a dose of 80 [40–100] mg daily. Conclusions. The median supply of iron (about 14 mg) and vitamin C (80 mg) in iron-containing multivitamin–multimineral preparations offered on Amazon platforms in Western and Southern Europe falls below that currently recommended for iron deficiency in review articles, namely 100 mg of iron and 500 mg of vitamin C per day. The iron supply of iron-containing multivitamin–multimineral preparations falls also below the dose of 30–60 mg advocated to prevent iron deficiency in menstruating women.

## 1. Introduction

Iron plays a central role in cellular processes, such as oxygen transport, respiration, metabolism, defense, and signaling, owing to its electron transfer abilities in redox reactions within proteins like heme and iron–sulfur clusters [1,2,3]. Excess “free” iron, however, can generate harmful free radicals, damaging deoxyribonucleic acid, proteins, and lipids. To balance essential iron functions and prevent toxicity, the human body employs homeostatic mechanisms to precisely regulate iron metabolism [1,2,3]. Hepcidin, the hormone regulating iron stores, inhibits its intestinal absorption, and promotes its release from recycling macrophages [3,4]. Iron deficiency suppresses hepcidin production to enhance its availability, while iron overload and inflammation stimulate hepcidin production to counter toxicity [1,2,3].

Body iron is strictly conserved. Healthy adults lose only about 1.0 to 1.5 mg (<0.5‰) of their 35 to 45 mg of iron per kilogram of body weight each day [1,2,3]. In Western and Southern Europe, with a dietary iron intake of 12 mg in females and 16 to 18 mg per day in males, the amount of absorbed iron is only 1.0 to 2.0 mg [3,5,6,7]. However, iron from animal sources is absorbed better than iron from plant sources [3,5,7].

Consequently, iron deficiency (without or with anemia) is a common nutrient deficiency worldwide. The estimated prevalence of overt iron deficiency approximates 20% in pregnant women, 15% in non-pregnant women, 10% in the elderly population, and 5% in men [1,2,3].

In the United States of America, the recommended dietary allowance for iron is 8 mg per day for men and postmenopausal women. It is 18 mg for women of childbearing age and 27 mg for pregnant women [3,5,7]. In Europe, the recommended allowances slightly vary: 8–11 mg for men and postmenopausal women, 15–18 for women of childbearing age, and 27 mg for pregnant women [3,5,7].

Oral iron supplementation is recommended to prevent iron deficiency in at-risk populations and particularly to treat iron deficiency. The World Health Organization recommends 30–60 mg of elemental iron daily to prevent iron deficiency in menstruating women [3]. On the other hand, for stable subjects with iron-deficiency anemia, the suggested daily dose is at least 100 mg of elemental iron [5,6]. Among the myriad of preparations on the market, ferrous (Fe^2+^) iron sulfate is the most frequently prescribed [3,5,6]. Gluconate and fumarate are also effective.

The addition of vitamin C, also known as ascorbic acid (usually 500 mg daily), may improve iron bioavailability, above all when ferric (Fe^3+^) preparations are prescribed [3,6,7]. In the United States, the suggested daily intake of vitamin C is 90 mg for men and 75 mg for women, 85 mg for pregnant women, and 120 mg for breastfeeding women [3,5,7]. In Europe, it is marginally higher: 110 mg for men, 95 mg for women, 100 mg for pregnant women, and 125 for breastfeeding women [3,5,7].

In high-income countries, multivitamin–multimineral supplements that can be purchased without medical prescription became available in the 1940s [8]. Online shopping of non-prescription multivitamin–multimineral supplements has tremendously increased over the recent years. Numerous multivitamin–multimineral supplements include iron and occasionally vitamin C. Nevertheless, it remains uncertain whether these online-sold preparations provide the daily iron amounts recommended to prevent deficiency in menstruating women, or the doses recommended for treating deficiency.

Amazon is a large and popular company that focuses, among other things, on electronic commerce of multivitamin–multimineral supplements. This labeling analysis aims to furnish insights into the quantification of iron (and vitamin C) content within multivitamin–multimineral merchandise that is available for commercialization through Amazon marketplaces across Western and Southern Europe.

## 2. Materials and Methods

### 2.1. Search Strategy

We surveyed multivitamin products for adult use on four Amazon marketplaces: amazon.de^®^, amazon.es^®^, amazon.fr^®^, and amazon.it^®^. The search through Amazon websites was carried out over the first three weeks of November 2023 (from 6 November to 24 November 2023). Two researchers conducted the research in duplicate, employing the search bar of each of the four marketplaces, as previously described [9]. The search used the terms “Multivitamins with iron” adapted to the languages of the four Amazon marketplaces.

A product was considered an iron-containing multivitamin–multimineral supplement if two or more vitamins or minerals were named in addition to iron (with or without vitamin C). The composition of each product obtained with the search was screened to ascertain the content of elemental iron, in either Fe^2+^ or Fe^3+^ state of oxidation, and vitamin C. Effort was made to determine the iron and vitamin C content in each product using the information declared on the online label of the product by the producer. After the selection of the eligible preparations, those without any information on the iron amount or state of oxidation were excluded. Preparations explicitly commercialized for newborns, infants, and children were also excluded. Finally, preparations specifically marketed for women were incorporated into the study and subjected to separate sub-analysis.

To determine the daily iron supply provided by an individual preparation, when a dose range was recommended (e.g., 1 to 3 pills per day), the highest amount was recorded. Preparations containing Fe^2+^ and preparations containing Fe^3+^ were recorded separately. Data regarding other minerals or vitamins contained in the preparation were also collected. Controversies in product selection and data extraction were solved by consensus. One author entered the extracted information into a designated database, while another author checked the accuracy of the data entry.

### 2.2. Statistical Analysis

Categorical variables are presented as counts and were evaluated using the Fisher exact test. The D’Agostino–Pearson omnibus test for normality revealed that continuous variables did not conform to a Gaussian distribution. Continuous data are therefore shown as median and interquartile range. For analysis, the nonparametric Mann–Whitney–Wilcoxon U test and the Kruskal–Wallis H test, followed by the Dunn’s post hoc multiple comparison, were applied [10,11,12]. A two-sided *p*-value below 0.05 was deemed statistically significant. The statistical analyses were carried out using GraphPad Prism for Macintosh version 10.2.3 (GraphPad Software, San Diego, CA, USA).

## 3. Results

We identified 312 iron-containing multivitamin–multimineral preparations sold by Amazon marketplaces. After excluding 14 preparations due to a lack of information regarding the oxidation state of the iron content, we analyzed the remaining 298 preparations, as summarized in Table 1. Specifically, 153 preparations were sourced from amazon.de^®^, 68 from amazon.fr^®^, 54 from amazon.it^®^, and 23 from amazon.es^®^ (Table 1).

**Figure 1:**
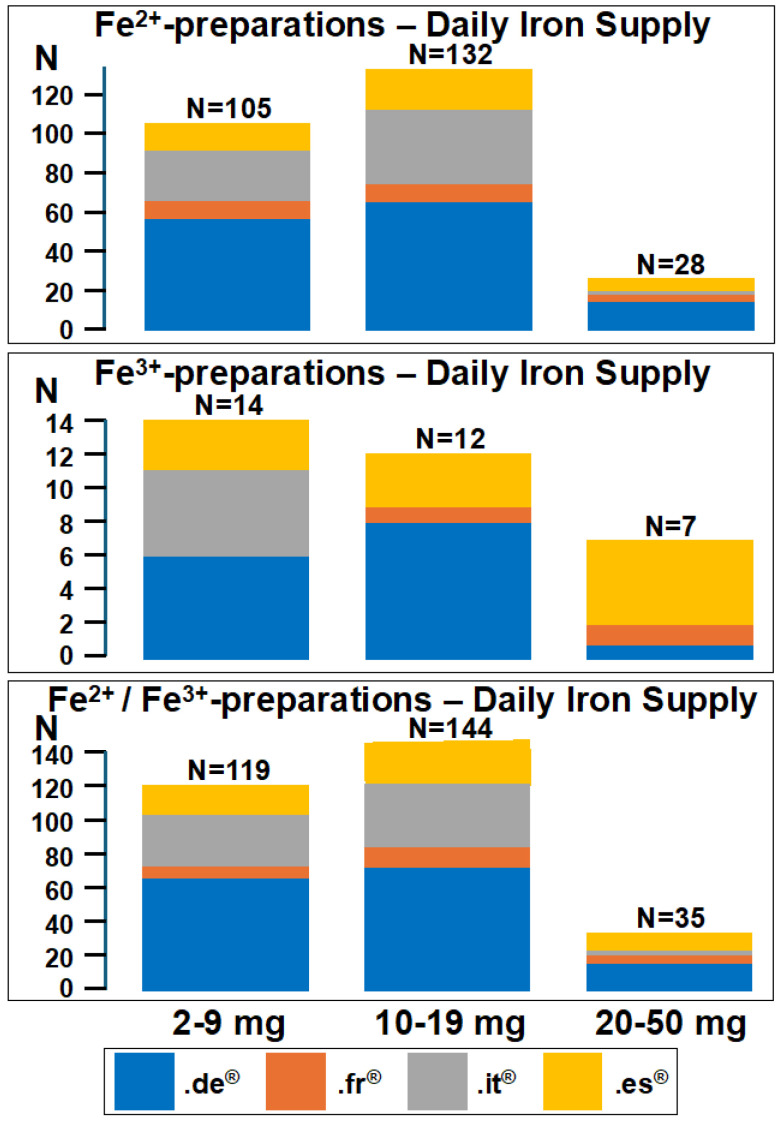
Daily iron supply provided by 298 iron-containing multivitamin–multimineral preparations sold by amazon.de^®^, amazon.fr^®^, amazon.it^®^, and amazon.es^®^. The preparations are subdivided into three different groups according to the suggested daily dose of iron, namely 2–9 mg, 10–19 mg, and 20–50 mg daily. Ferrous (Fe^2+^) iron- and ferric (Fe^3+^) iron-containing preparations are presented separately.

About 90% of the preparations contained Fe^2+^, without statistically significant difference among the four marketplaces (Table 1 and Figure 1).

Among the 265 Fe^2+^ preparations, 229 were supplemented with vitamin C (amazon.de^®^: 114 out of 138; amazon.fr^®^: 57 out of 63; amazon.it^®^: 39 out of 43; and amazon.es^®^: 19 out of 21). Among the 33 Fe^3+^ preparations, 24 were supplemented with vitamin C (amazon.de^®^: 11 out of 15; amazon.fr^®^: 4 out of 5; amazon.it^®^: 8 out of 11; and amazon.es^®^: 1 out of 2). The iron supply provided by the multivitamin–multimineral preparations fell below 20 mg daily in most of the multivitamin–multimineral preparations, as shown in Figure 1. The amount of vitamin C provided by Fe^2+^ preparations (80 [80–120] mg) was significantly (*p* < 0.0001) higher than that provided by Fe^3+^ preparations (55 [36–80] mg). The vitamin C over iron ratio was similar among the four marketplaces both for Fe^2+^ and Fe^3+^ products. Interestingly, this ratio was significantly (*p* = 0.0037) higher in Fe^2+^ (8.6 [5.7–20]) than in Fe^3+^ (5.7 [2.2–11]) preparations. Table 2 provides details regarding the presence of elements other than iron and vitamins other than vitamin C in the 298 iron-containing multivitamin–multimineral preparations.

Approximately 83% of these preparations contained at least one element other than iron, while 87% included at least one vitamin other than vitamin C. The number of elements other than iron provided by preparations sold by amazon.fr^®^ was slightly higher (*p* = 0.0497) than in those provided by amazon.it^®^.

Fifty-one preparations (amazon.de^®^, n = 16; amazon.it^®^, n = 9; amazon.fr^®^, n = 18; and amazon.es^®^, n = 8) were explicitly recommended for consumption by women. Out of these, 44 preparations contained Fe^2+^, while the other 7 contained Fe^3+^. The elemental iron content provided by these preparations appeared to be almost identical (14 [7–14] mg daily) to that of the remaining 247 preparations (14 [5–14] mg daily; *p* = 0.555). Forty-six out of the fifty-one preparations intended for women also featured vitamin C supplementation. The vitamin C supply was almost identical in preparations intended for women (80 [80–90] mg) and in those intended for the general population (80 [40–100] mg; *p* = 0.389).

## 4. Discussion

Multivitamin–multimineral supplements lack universally accepted scientific, regulatory, or market-specific definitions [8,13,14]. Nevertheless, in the United States, approximately one-third of adults incorporate these supplements into their dietary regimens [15], and a similar pattern is observed in numerous European nations [16,17,18]. A preliminary examination of Google Trend data conducted by the authors of this paper reveals a substantial upsurge in consumer interest in multivitamin and multimineral preparations within countries such as France, Germany, Italy, Switzerland, the United Kingdom, and the United States, with search volume tripling over the past decade [9]. These supplements are frequently consumed during the winter months, hoping to mitigate the risk of acute respiratory diseases [19]. Many individuals resorted to their usage to enhance immune responses and mitigate effects of the coronavirus disease 2019 pandemic [20].

We undertook an examination of the iron and vitamin C content in nearly 300 iron-containing multivitamin–multimineral preparations available for sale on Amazon platforms in Western and Southern Europe (amazon.de^®^, amazon.fr^®^, amazon.it^®^, and amazon.es^®^). The findings reveal that the elemental iron provision of these preparations is predominantly below 20 mg per day, without relevant disparities observed among the four marketplaces. Furthermore, approximately 90% of the preparations contain Fe^2+^. Finally, slightly over 85% of these preparations are enriched with vitamin C, with a median daily supply of approximately 80 mg. The median supply of elemental iron (about 14 mg) and vitamin C (80 mg) in iron-containing multivitamin–multimineral preparations offered on Amazon platforms in Germany, France, Italy, and Spain falls appreciably below that currently recommended, namely 100 mg of elemental iron per day [3,5,6] and 500 mg of vitamin C [3]. A mg/mg ratio of vitamin C to iron for optimal absorption is at least three times the amount of iron. Therefore, the vitamin C to iron ratios in supplements available on Amazon marketplaces might be suitable [3].

The iron supply of iron-containing multivitamin–multimineral preparations also falls below the dose of 30–60 mg advocated to prevent iron deficiency in menstruating women [3].

The Fe^3+^ formulations contained significantly less vitamin C compared to the Fe^2+^ ones. Given that Fe^3+^ is less effectively absorbed in the intestine than Fe^2+^, and since vitamin C can convert Fe^3+^ to Fe^2+^, thereby enhancing iron bioavailability, the lower amount of vitamin C in the Fe^3+^ formulations appears paradoxical [21]. The content of both iron and vitamin C in preparations tailored for women does not differ from those designed for the general population. This is counterintuitive considering the increased risk of iron-deficiency anemia among fertile women. Supplementation with vitamin C, which lowers urine pH, may increase the risk of kidney stones in men taking 500 mg or more daily [22]. However, the vitamin C content in the analyzed products was significantly much lower.

Oral iron is generally considered safe for over-the-counter availability. However, its utilization is linked to a range of gastrointestinal adverse effects, such as nausea (occasionally also vomiting), constipation (less frequently diarrhea), flatulence, and post-meal fullness [5,6,7,23]. The occurrence and severity of these side effects are directly related to the quantity of elemental iron administered, with lower elemental iron doses being associated with less adverse effects. Consequently, the iron in multivitamin–multimineral supplements is generally kept to a modest level.

Recent data have questioned the currently recommended and long-established approach of administering at least 100 mg of Fe^2+^ daily in two to three divided doses to treat iron deficiency [7,24,25,26,27]. These data suggest that lower doses, like 15 to 20 mg of elemental iron once daily, can be equally effective and better tolerated [7,24,25,26,27]. It is assumed that the effectiveness of low-dose iron once daily is likely due to the saturation of intestinal iron absorption [28], where a single iron dose can hinder the absorption of subsequent doses for the rest of the day [6,7,26,28]. This could be linked to a rapid rise in hepcidin level following iron intake [26,28].

This cross-sectional labeling analysis has some limitations. The analysis focused on iron and vitamin C in four European Amazon platforms, potentially limiting generalizability. We did not verify whether the iron and vitamin C on the label match the supplement’s content. Next, companies like iHerb^®^ and Alibaba.com^®^ also sell dietary supplements online. Additionally, multivitamin use is common in other regions, such as Saudi Arabia. Finally, supermarkets, grocery stores, and pharmacies also offer dietary supplements. Therefore, our analysis does not provide a whole picture of the over-the-counter commerce of multivitamin–multimineral preparations that contain iron. The current study, however, offers a useful knowledge basis and it might well anticipate the likely results of such studies, which we expect to be very similar. This study has at least three relevant strengths. First, several Amazon platforms were searched and compared. Second, although the analysis was focused on iron, we also separately considered the content related to other elements and vitamins. 

## 5. Conclusions

This study examined the iron and vitamin C content in iron-enriched multivitamin–multimineral preparations sold on various Amazon platforms in Western and Southern Europe. The findings reveal that most preparations contain less than 20 mg of elemental iron per day, with no significant differences among marketplaces. A total of 89% of the preparations contain Fe^2+^ iron, and approximately 91% are enriched with around 80 mg of vitamin C per day. It is worth noting that both the elemental iron and vitamin C levels in these preparations fall below recommended levels from reputable scientific sources. Interestingly, recent data suggest that lower iron doses may be similarly effective and better tolerated. Physicians often, and rightly so, tend to view the use of dietary supplements purchased online (or supermarkets, grocery stores, and pharmacies) as superfluous. However, this is a reality that cannot be ignored. The data from our label analysis are, in our view, useful for physicians who need to prescribe iron (with or without vitamin C) to patients with a deficiency who are already using an iron-containing dietary supplement.

## Figures and Tables

**Table 1:** Characteristics of 298 iron-containing multivitamin–multimineral products.

			All	Amazon.de^®^	Amazon.fr^®^	Amazon.it^®^	Amazon.es^®^	*p*-Value
**Ferrous (Fe^2+^) Iron,** n			265	138	63	43	21	
	Daily Iron Dose, mg		14 [5–14]	14 [5–14]	14 [5–14]	14 [6–14]	10 [7–15]	0.5993
	Vitamin C							
		*N*	229 (91)	114 (91)	57 (93)	39 (83)	19 (95)	0.3705
		Daily Vitamin C Dose, mg	80 [80–120]	80 [80–120]	80 [80–100]	90 [80–110]	90 [80–158]	0.4676
		Vitamin C over Iron Ratio, mg/mg	8.6 [5.7–20]	9.8 [5.7–20]	7.1 (5.7–19]	8.0 (5.7–19)	14 (7.4–21)	0.6069
**Ferric (Fe^3+^) Iron, n**			33	15	5	11	2	
	Daily Iron Dose, mg		14 [5–16]	14 [6–14]	6 [2–7]	14 [10–29]	14, 42 *	0.069
	Vitamin C							
		*N*	24 (76)	11 (73)	4 (80)	8 (57)	1 (50)	0.9358
		Daily Vitamin C Dose, mg	55 [36–80]	80 [60–90]	12, 40, 40, 80 *	40 [24–61]	55 *	0.1916
		Vitamin C over Iron Ratio, mg/mg	5.7 [2.2–11]	8.6 [4.8–13]	5.7, 6.0, 6.2, 11	3.9 [1.4–5.4]	1.3	0.1100

Results are given as frequency (often with percentage) or as median (with interquartile range]. If <6 data were available, individual values are presented. The vitamin C over iron ratio was significantly (*p* = 0.0037) higher in Fe^2+^ (8.6 [5.7–20]) than in Fe^3+^ (5.7 [2.2–11]) preparations. * individual values.

**Table 2:** Elements other than iron and vitamins other than vitamin C contained in iron-containing multivitamin–multimineral products.

			Market Places				
		All	Amazon.de^®^	Amazon.fr^®^	Amazon.it^®^	Amazon.es^®^	*p*-Value
**N**		298	153	68	54	23	
**Elements other than iron**							
	0	52 (17)	21 (14)	8 (12)	20 (37)	3 (13)	0.0497
	1–5	89 (30)	56 (37)	15 (22)	9 (17)	9 (39)	
	6–10	139 (47)	66 (43)	41 (60)	22 (41)	10 (44)	
	11–13	18 (6)	10 (6)	4 (6)	3 (5)	1 (4)	
**Vitamins other than vitamin C**							
	0	40 (13)	21 (14)	6 (9)	11 (20)	2 (9)	0.0965
	1–5	45 (15)	29 (19)	3 (4)	11 (20)	2 (9)	
	6–10	55 (19)	27 (17)	16 (24)	4 (8)	8 (34)	
	11–13	158 (53)	76 (50)	43 (63)	28 (52)	11 (48)	

## Data Availability

The original contributions presented in the study are included in the article; further inquiries can be directed to the corresponding author.
